# Immune checkpoint inhibitor-associated diabetes mellitus: an overlooked immune-related adverse event—two case reports and a literature review

**DOI:** 10.3389/fimmu.2026.1831879

**Published:** 2026-05-08

**Authors:** Mengjia He, Zhenru Zheng, Xiaofan Guo, Gang Cheng, Shuang Zhang, Jinlong Liu, Jing Zhao

**Affiliations:** 1 Department of Oncology, Hebei General Hospital, Shijiazhuang, China; 2 Graduate School, Hebei Medical University, Shijiazhuang, China; 3 Department of Oncology, Hebei University of Chinese Medicine Hebei Provincial Hospital of Traditional Chinese Medicine, Shijiazhuang, China; 4 Graduate School, Hebei North University, Zhangjiakou, China

**Keywords:** adverse events, diabetes mellitus, diabetic ketoacidosis, immune checkpoint inhibitor, PD-1, PD-L1

## Abstract

Immune checkpoint inhibitor-associated diabetes mellitus (ICI-DM) is a relatively uncommon manifestation of immune-related adverse events. However, it often presents with diabetic ketoacidosis (DKA) at onset, which may lead to severe and potentially life-threatening complications. Therefore, early recognition and treatment are essential. This condition typically occurs within the first three months after initiation of immunotherapy, whereas delayed onset after treatment discontinuation has been infrequently reported. In this study, Case 1 developed diabetic ketoacidosis 9 weeks after discontinuation of serplulimab (approximately 19 months after treatment initiation), representing a rare delayed-onset presentation. Case 2 developed hyperglycemia after 23 weeks of envafolimab therapy and exhibited preserved islet function without DKA, suggesting a possible early-stage or type 2 diabetes-like phenotype of ICI-related dysglycemia. Both patients achieved glycemic control with exogenous insulin therapy. Immunotherapy was discontinued in Case 1, whereas Case 2 continued treatment with satisfactory glycemic control. This study summarizes the pathogenesis, clinical characteristics, management, and prognosis of ICI-DM, highlighting its heterogeneity and the importance of early detection of ICI-related dysglycemia. Early and continuous glucose monitoring during and after immunotherapy is essential, and ongoing follow-up after treatment discontinuation remains warranted.

## 1 Introduction

Immune checkpoint inhibitors (ICIs) enhance antitumor immunity by blocking tumor-mediated immune evasion and restoring immune surveillance. They represent a major breakthrough in cancer therapy and are now widely used in the treatment of various malignancies, including malignant melanoma and non-small cell lung cancer. However, excessive immune activation induced by ICIs can result in damage to normal tissues, leading to immune-related adverse events (irAEs), such as pneumonitis, colitis, hepatitis, and a spectrum of endocrine disorders (1). Among endocrine irAEs, thyroid and pituitary dysfunction are relatively common, whereas immune checkpoint inhibitor-associated diabetes mellitus (ICI-DM) is rare, with an incidence of approximately 1–2% (2). Despite its low incidence, ICI-DM is clinically significant because it often presents acutely and may rapidly progress to life-threatening diabetic ketoacidosis (DKA). Notably, blockade of programmed cell death protein-1 (PD-1) or its ligand (PD-L1) is associated with a higher risk of ICI-DM compared with cytotoxic T-lymphocyte antigen-4 (CTLA-4) inhibition (3). Therefore, heightened vigilance is warranted during treatment with PD-1/PD-L1 inhibitors. Although awareness of ICI-DM has increased, early diagnosis remains challenging due to its non-specific presentation. Here, we report two cases of ICI-related dysglycemia following PD-1/PD-L1 inhibitor therapy, illustrating distinct clinical phenotypes to improve recognition and management.

## 2 Case presentation

### 2.1 Case 1

**Figure 1:**
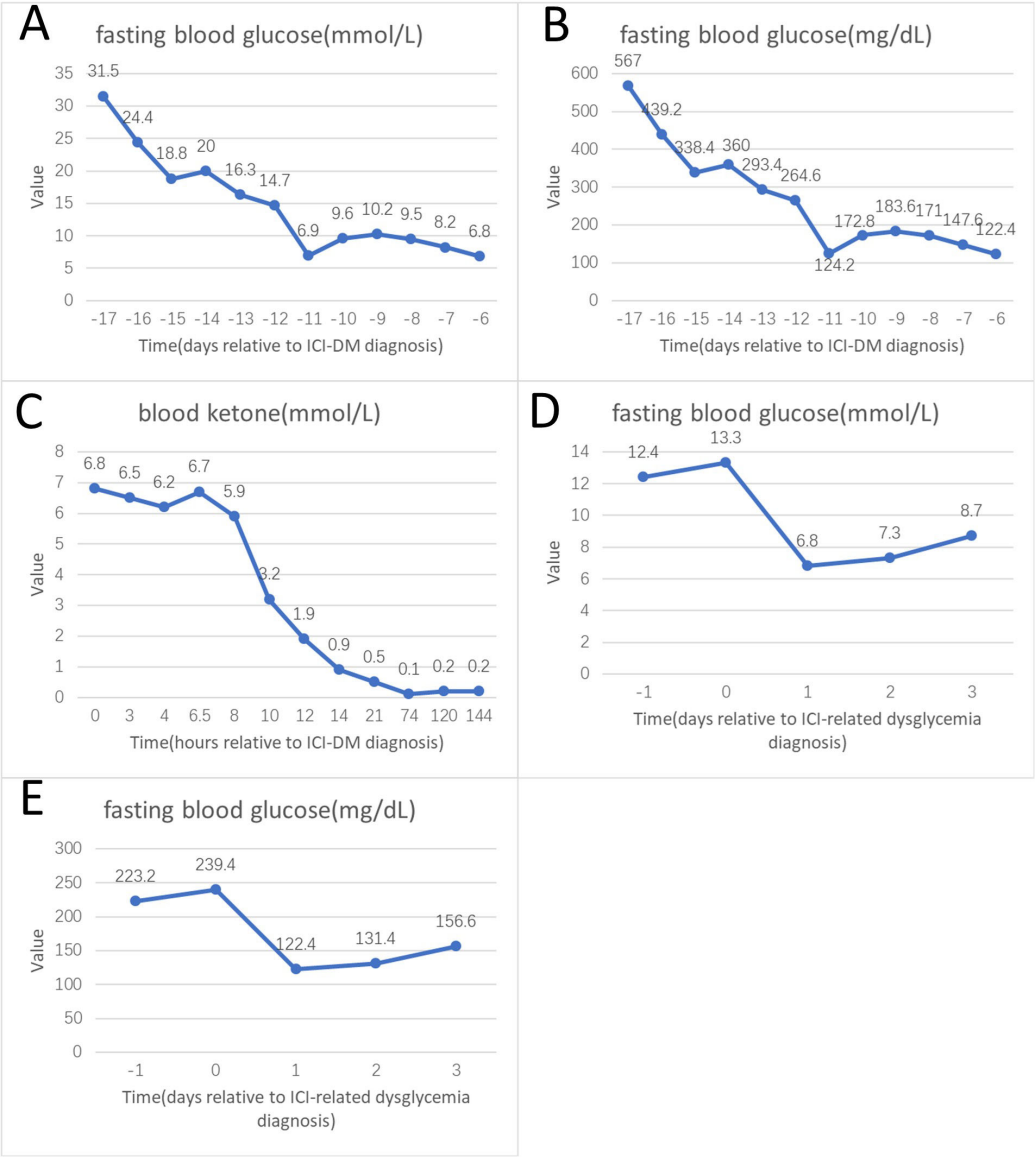
Longitudinal changes in blood glucose in two patients and blood ketone levels in Case 1 with ICI-DM. **(A, B)** Fasting blood glucose levels in Case 1 during the first episode of diabetic ketoacidosis (DKA), presented in mmol/L **(A)** and mg/dL **(B)**. **(C)** Blood ketone levels in Case 1 during the second episode of DKA. **(D, E)** Fasting blood glucose levels in Case 2 at the time of hyperglycemia onset, presented in mmol/L **(D)** and mg/dL **(E)**. Time is expressed relative to the diagnosis of ICI-DM in Case 1 **(A–C)** and to the diagnosis of ICI-related dysglycemia in Case 2 **(D, E)**. Days are shown in panels **(A, B, D, E)**, whereas hours are shown in **(C)** Day 0 corresponds to the time of diagnosis in each case. No ketone data were available for Case 2.

**Figure 2:**
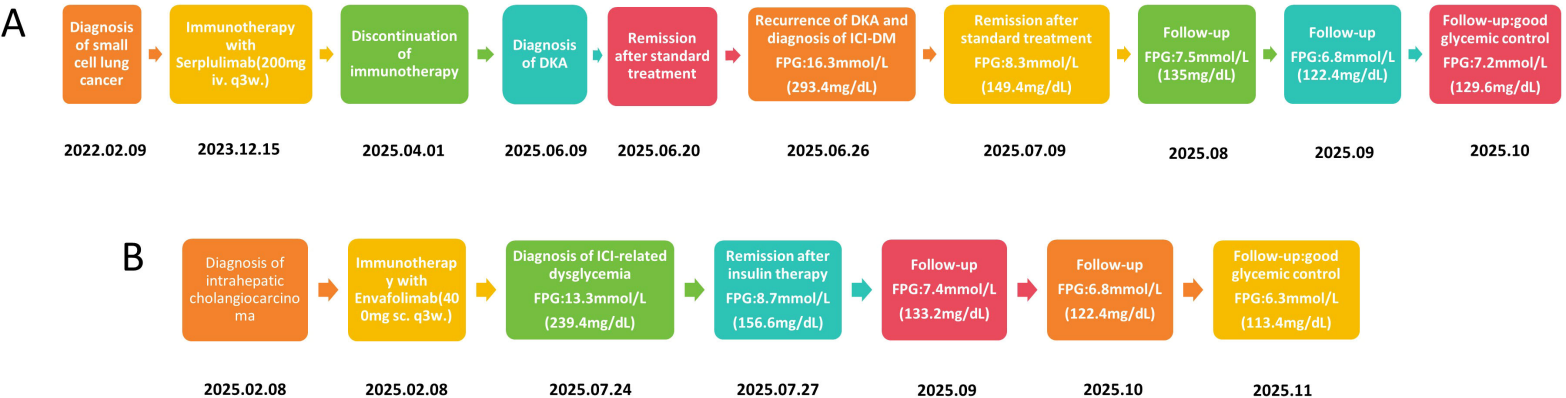
Clinical course and longitudinal glycemic changes in two patients. **(A)** Case 1 **(B)** Case 2. Abbreviations: i.v., intravenous injection; q3w, every 3 weeks; DKA, diabetic ketoacidosis; s.c, subcutaneous injection; FPG, fasting plasma glucose.

In February 2022, a 66-year-old woman presented with intermittent cough and unexplained shortness of breath. She had no prior history of diabetes mellitus, no family history of diabetes, and was not exposed to corticosteroid therapy at any time from admission until the onset of hyperglycemia. Baseline laboratory evaluation prior to initiation of immunotherapy demonstrated normal glycemic status, with a fasting plasma glucose (FPG) of 5.3 mmol/L (95.4 mg/dL), glycated hemoglobin (HbA1c) of 5.5%, and a fasting C-peptide level of 1.8 ng/mL (reference range, 0.2–4.0 ng/mL), with no clinical evidence of dysglycemia. Chest computed tomography (CT) revealed a central-type mass in the left lower lobe measuring approximately 59 × 56 × 74 mm, highly suggestive of lung carcinoma. The lesion involved the left lower pulmonary artery and vein, compressed the left atrium with indistinct margins, and invaded the pleura. A slightly enlarged mediastinal lymph node was noted to the left of the aortic arch, accompanied by minimal left-sided pleural effusion. No distant metastases were identified on brain magnetic resonance imaging (MRI), abdominal and pelvic CT, or whole-body bone scan. Bronchoscopy demonstrated exophytic stenosis at the left lower lobe bronchial opening, and a biopsy was obtained. Histopathological examination confirmed small cell carcinoma. The final diagnosis was extensive-stage small cell lung carcinoma (T4NxM1a, stage IVA). The patient received 22 cycles of immunotherapy with serplulimab (200 mg per cycle). Due to disease progression, the treatment regimen was subsequently changed to paclitaxel (300 mg) in combination with anlotinib (8 mg), achieving a partial response (PR) after two cycles. Nine weeks after discontinuation of serplulimab, the patient developed acute symptoms, including dry mouth, nausea, fatigue, polyuria, and general malaise. Initial laboratory findings showed a serum glucose level of 31.55 mmol/L (567.9 mg/dL), pH of 7.12, serum bicarbonate of 4.80 mmol/L, partial pressure of carbon dioxide of 15.22 mmHg, base excess of -22.35 mmol/L, anion gap of 30.70 mmol/L, urine ketones 3+, urine glucose 4+, and HbA1c of 7.6%. The patient was diagnosed with DKA and treated with intravenous fluid resuscitation, continuous insulin infusion, correction of electrolyte imbalance, and resolution of ketosis. After resolution of DKA, the patient was initially managed under a provisional diagnosis of type 2 diabetes mellitus, with subcutaneous insulin aspart before meals, insulin glargine at bedtime, and oral antidiabetic agents (retagliptin and henagliflozin). Approximately one week after discharge, the patient was readmitted with recurrent symptoms of dry mouth, polydipsia, polyuria, fatigue, nausea, and vomiting. Laboratory findings again confirmed DKA, with a serum glucose level of 16.3 mmol/L (293.4 mg/dL), pH of 7.07, serum bicarbonate of 3.00 mmol/L, partial pressure of carbon dioxide of 10.59 mmHg, base excess of −24.90 mmol/L, anion gap of 30.90 mmol/L, and blood ketones of 6.8 mmol/L. She was managed according to standard DKA treatment protocols. Serial glucose and ketone measurements are shown in **Figures 1A–C**. Testing for diabetes-related autoantibodies, including glutamic acid decarboxylase antibody (GADA), insulin autoantibody (IAA), islet cell antibody (ICA), and islet antigen-2 antibody (IA-2A), was negative. An oral glucose tolerance test (OGTT) with insulin and C-peptide measurements demonstrated profound β-cell dysfunction, with markedly impaired endogenous insulin secretion (**Table 1A**). Given the recurrence of DKA shortly after discharge while receiving oral antidiabetic therapy, particularly in the context of SGLT-2 inhibitor use, the initial diagnosis was reconsidered. On June 26, 2025, the diagnosis was revised to ICI-DM. Oral antidiabetic agents were immediately discontinued, and intensive insulin therapy was initiated. During the follow-up period, the patient maintained good glycemic control, while C-peptide levels remained persistently low, consistent with ongoing β-cell dysfunction without evidence of recovery (**Figure 2A**).

**Table 1A:** OGTT with insulin and C-peptide measurements for case 1.

Parameter	0	30min	60min	120min	180min
Blood glucose (mmol/L[mg/dL])	17.07 [307.26]	12.40 [223.2]	20.38 [366.84]	26.80 [482.4]	28.98 [521.64]
Insulin(μU/mL)	2.688	14.924	13.900	12.007	12.281
C-peptide(ng/mL)	<0.200	<0.200	<0.200	<0.200	<0.200

### 2.2 Case 2

In February 2025, a 62-year-old man presented with a 2-year history of right upper abdominal discomfort, which had worsened over the preceding two months. He had no prior history of diabetes mellitus, no family history of diabetes, and was not exposed to corticosteroid therapy at any time from admission until the onset of hyperglycemia. Baseline laboratory evaluation prior to initiation of immunotherapy (January 2025) demonstrated normal glycemic status, with a fasting plasma glucose (FPG) of 5.4 mmol/L (97.2 mg/dL), glycated hemoglobin (HbA1c) of 5.6%, and a fasting C-peptide level of 2.1 ng/mL (reference range, 0.2–4.0 ng/mL), with no clinical evidence of dysglycemia. Hepatobiliary and pancreatic CT revealed intrahepatic cholangiocarcinoma with multiple intrahepatic metastases, accompanied by compression of the adjacent right hepatic vein and inferior vena cava. Multiple enlarged lymph nodes were noted in the hepatic hilum, pericholecystic region, and retroperitoneum, suggestive of metastatic disease. A nodular lesion in the right adrenal gland was also observed and considered metastatic. On February 8, 2025, an ultrasound-guided liver biopsy was performed. Histopathological examination confirmed invasive adenocarcinoma consistent with intrahepatic cholangiocarcinoma. The final diagnosis was intrahepatic cholangiocarcinoma (T2N1M1, stage IV). The patient received seven cycles of immunotherapy with envafolimab (400 mg per cycle). On July 17, 2025, he self-detected an elevated blood glucose level. Initial laboratory findings showed a serum glucose level of 13.01 mmol/L (234.18 mg/dL), partial pressure of carbon dioxide of 33.49 mmHg, pH of 7.42, serum bicarbonate of 21.20 mmol/L, urine glucose of 4+, urine ketones negative, and HbA1c of 9.5%, without evidence of diabetic ketoacidosis. Testing for diabetes-related autoantibodies was negative. An oral glucose tolerance test (OGTT) with insulin and C-peptide measurements demonstrated preserved C-peptide levels with an appropriate response to glucose stimulation, suggesting an insulin resistance phenotype (**Table 1B**). Insulin pump therapy was initiated, and blood glucose levels stabilized within 10 days (**Figure 1D, E**). The patient was subsequently transitioned to a subcutaneous insulin regimen consisting of insulin aspart before meals and insulin degludec at bedtime. During the follow-up period, the patient maintained satisfactory glycemic control, while C-peptide levels declined over time, suggesting a trend toward progressive β-cell dysfunction (**Figure 2B**).

**Table 1B:** OGTT with insulin and C-peptide measurements for case 2.

Parameter	0	30min	60min	120min	180min
Blood glucose(mmol/L)	17.07	NM	17.74	21.32	NM
C-peptide (ng/mL)	5.269	NM	6.510	6.643	NM

NM, not measured.

## 3 Discussion

ICI-DM is a distinct form of diabetes associated with immune checkpoint inhibition. Emerging evidence suggests that ICI-DM is clinically heterogeneous. Recent reports suggest that ICI-DM can be broadly categorized into several clinical phenotypes. The first and best-characterized phenotype is fulminant autoimmune type 1 diabetes mellitus (T1DM), characterized by abrupt onset, frequent presentation with DKA, relatively normal or only mildly elevated HbA1c levels, and markedly reduced or undetectable C-peptide levels. The second phenotype presents as a more gradually progressive hyperglycemia resembling type 2 diabetes mellitus (T2DM), with significantly elevated HbA1c levels but relatively preserved C-peptide levels. This form may reflect immune-mediated insulin resistance or inflammation-related metabolic dysregulation, potentially driven by cytotoxic lymphocyte activation or tumor-associated inflammatory responses. Additional reported phenotypes include diabetes secondary to autoimmune pancreatitis and ICI-associated lipodystrophy-related diabetes. Among these, the fulminant insulin-deficient phenotype is the best characterized, and Case 1 in our study is consistent with this phenotype (4–6).

Compared with conventional T1DM, ICI-DM typically occurs at an older age, has a lower incidence, presents more abruptly, and is associated with a lower prevalence of diabetes-related autoantibodies (7, 8). Approximately 30%–76% of patients present with DKA at onset (9), highlighting the severity of this condition.

The timing of ICI-DM onset is highly variable. A meta-analysis reported that 71% of cases occur within 90 days of immunotherapy initiation, typically after an average of 4.5 cycles of PD-1/PD-L1 inhibitor therapy. Onset is earlier in patients receiving combined CTLA-4 and PD-1 inhibitor therapy, occurring after approximately 2.7 cycles (3). Further studies indicate that combining ICIs with chemotherapy also increases the incidence of ICI-DM, with onset occurring approximately 2.5 cycles earlier than with monotherapy (4.5 cycles on average) (10). In contrast, delayed-onset cases are rare. In the present study, Case 1 developed diabetes 18.6 months after initiation of PD-1 inhibitor therapy and 70 days after treatment discontinuation, representing a delayed-onset presentation. A systematic PubMed literature search identified 12 previously documented cases of delayed-onset ICI-DM (**Table 2**). These cases had a median age of 60 years, a median duration of ICI therapy of 9 months, and a median interval from the last ICI administration to ICI-DM onset of 4 months (8, 10–19).

**Table 2:** Cases of delayed-onset ICI-DM. F, female; M, male; NSCLC, non-small cell lung cancer; NA, not applicable; HbA1c, glycated hemoglobin; -, negative; GADA, glutamic acid decarboxylase antibody; IA-2A, islet antigen-2 antibody; IAA, insulin autoantibody; ZnT8A, zinc transporter 8 antibody; ICA, islet cell antibody; NM, not mentioned; DKA, diabetic ketoacidosis; Grade 2 irH with IAD, grade 2 immune-related hypophysitis with isolated adrenocorticotropic hormone deficiency.

Case No.	Age(yr)	Sex	Cancer	ICI regimen	Duration of ICI therapy	Period from the last ICI administration to the onset of T1DM	Plasma glucose(mmol/L)	HbA1c(%)	Diabetes-associated antibodies	Discontinuation of immunotherapy	Fasting C-peptide(ng/mL)	DKA	History of diabetes	Complicated with other new-onset endocrine disorders
1^11^	73	F	Lung adenocarcinoma	Atezolizumab	2 months	4 months	53.4	7.3	-(GADA,IA-2A)	Yes	NM	Yes	NM	No
2^12^	59	M	Gastric cancer	Nivolumab	9 months	4 months	38.3	10.6	-(GADA,IA-2A,ZnT8)	Yes	0.55	Yes	No	No
3^13^	60	F	Renal cell carcinoma	Nivolumab and ipilimumab	2 months	6 months	18.3	6.5	-(GADA)	Yes	0.25	Yes	No	No
4^14^	51	M	Melanoma	Nivolumab and ipilimumab	9 months	2 months	46.4	7.9	-(GADA,IAA,IA-2A)	Yes	0.97	Yes	No	Grade 2 irH with IAD
5^15^	53	M	Gastric cancer	Nivolumab	66 months	NA	36	7.8	-(GADA,IAA,IA-2A,ICA,ZnT8A)	No	NM	Yes	No	No
6^16^	47	M	Nodular melanoma	Pembrolizumab	12 months	5 months	26.7	8.3	-(GADA,IA-2A)	Yes	0.49	Yes	No	No
7^10^	70	M	Small cell lung cancer	Serplulimab	24 months	NA	27.2	11.9	-(GADA,IA-2A,IAA,ZnT8A)	No	0.33	Yes	No	Thyroid dysfunction
8^10^	64	M	Small cell lung cancer	Serplulimab	12 months	NA	>33	9.6	-(GADA,IA-2A,IAA,ZnT8A)	No	0.03	Yes	No	Thyroid dysfunction
9^17^	60	M	Gastric adenocarcinoma	Sintilimab	3 months	4 months	42.01	7.5	-(GADA,IAA,ICA)	Yes	<0.02	Yes	No	Thyroid dysfunction
10^8^	74	M	NSCLC	Nivolumab	6 months	4 months	41	10.6	-(GADA),IAA was borderline	Yes	0.03	Yes	No	No
11^18^	53	M	Esophageal adenocarcinoma	Tislelizumab	6 months	11 months	13.56	NM	-(GADA,IAA,ICA)	Yes	0.06	Yes	Yes	Thyroid dysfunction
12^19^	60	M	Melanoma	Pembrolizumab	12 months	2 months	15.4	6.9	-(GADA,ICA,IA-2A)	Yes	NM	No	No	Thyroid dysfunction
Our case 1	66	F	Small cell lung cancer	Serplulimab	19 months	2 months	31.55	7.6	-(GADA,IAA,ICA,IA-2A)	Yes	<0.02	Yes	No	No

The pathogenesis of ICI-DM remains incompletely understood but is thought to be primarily driven by breakdown of PD-1/PD-L1–mediated immune tolerance and subsequent T cell–mediated β-cell destruction. Blockade of this pathway promotes activation and expansion of autoreactive T cells, particularly cytotoxic CD8⁺ T cells, which infiltrate pancreatic islets and accelerate β-cell loss (20, 21). Recent reviews and clinical studies support this mechanism and suggest that inflammatory signaling may further amplify β-cell injury, contributing to the fulminant insulin-deficient phenotype observed in many patients (20). Preclinical studies provide additional biologic support for this model. In non-obese diabetic mouse models, PD-1 or PD-L1 blockade rapidly precipitates autoimmune diabetes, indicating that the PD-1/PD-L1 axis is a key regulator of islet immune tolerance (21). Delayed-onset ICI-DM after treatment discontinuation may be explained by the sustained immunologic effects of checkpoint blockade. PD-1 receptor occupancy and immune activation can persist well beyond the last dose, and delayed immune-related adverse events have been increasingly recognized. Accordingly, a subclinical phase of ongoing β-cell destruction may continue after drug withdrawal until overt hyperglycemia or diabetic ketoacidosis develops (22, 23).

The diagnostic criteria for ICI-DM remain unclear. The latest NCCN guidelines propose diagnostic criteria for ICI-DM, recommending fasting blood glucose >11.1 mmol/L or random blood glucose >13.9 mmol/L, or a history of T2DM with fasting/random blood glucose >13.9 mmol/L. Testing for autoantibodies and C-peptide levels should be considered to assess and classify ICI-DM (24). Glutamic acid decarboxylase antibody (GADA) is among the more prevalent and persistent diabetes-related autoantibodies. Reports indicate that the positivity rate for diabetes-related autoantibodies in ICI-T1DM patients ranges approximately from 26 to 56%, lower than that observed in T1DM patients (25). Patients with positive antibodies (particularly GADA positivity) exhibit a shorter median time from initiation of ICI therapy to diabetes onset (7). C-peptide is a widely used marker of endogenous insulin secretion. In the majority of patients, islet function deteriorates rapidly at onset, with 85–93% exhibiting low or undetectable C-peptide levels at presentation, and spontaneous recovery is uncommon. This finding reflects substantial impairment of endogenous insulin secretion (26).

Case 1 exhibited classical features of fulminant ICI-DM, including abrupt onset, recurrent DKA, and severe insulin deficiency. Notably, hyperglycemia developed after discontinuation of immunotherapy and was not initially recognized as ICI-DM. The patient was provisionally misdiagnosed with T2DM and treated with oral antidiabetic agents, including an SGLT-2 inhibitor. The subsequent recurrence of DKA strongly suggested an insulin-deficient state rather than insulin resistance. SGLT-2 inhibitors are known to increase the risk of diabetic ketoacidosis, including euglycemic DKA, particularly in insulin-deficient conditions (27–29). Therefore, this clinical course further supports that the underlying pathophysiology was consistent with insulin-dependent ICI-DM rather than T2DM and highlights the importance of early recognition of ICI-DM and cautious use of oral glucose-lowering agents in this setting.

In contrast, Case 2 exhibited a non-fulminant phenotype at presentation. Preserved C-peptide levels, absence of DKA, and OGTT findings suggestive of insulin resistance were atypical of classical fulminant ICI-DM. The differential diagnosis included steroid-induced hyperglycemia and previously unrecognized T2DM. However, the lack of corticosteroid exposure, near-normal baseline glycemic parameters before immunotherapy (FPG 5.4 mmol/L and HbA1c 5.6%), and the temporal association with PD-L1 inhibitor therapy argue against these possibilities, although they cannot be fully excluded. Importantly, longitudinal follow-up demonstrated a progressive decline in C-peptide levels, supporting evolving β-cell dysfunction over time. Taken together, these findings favor a diagnosis of ICI-related dysglycemia with a T2DM-like phenotype at onset rather than classical fulminant ICI-DM. This case may represent an early, pre–islet failure phase of ICI-related diabetes that is likely underrecognized in clinical practice, as reported cases are often identified only after overt metabolic decompensation, particularly DKA (30, 31). The initial insulin resistance observed in this patient may have been multifactorial, reflecting systemic inflammation, tumor-related metabolic stress, and compensatory hyperinsulinemia during early immune-mediated β-cell injury (20, 31).

Management of ICI-DM generally requires insulin therapy, as the majority of patients develop profound and typically persistent insulin deficiency (32). Both patients in the present study achieved satisfactory glycemic control with insulin-based treatment. Although long-term insulin dependence is typical, recovery of endogenous insulin secretion may occur in rare cases. For example, Okubo et al. (33) reported a patient with ICI-DM who demonstrated improved insulin secretion on glucagon stimulation testing after 6 months of insulin therapy and was subsequently able to discontinue insulin treatment (33). Because ICI-DM often presents abruptly and frequently with DKA, early recognition is essential. Prompt measurement of blood and urine ketones is particularly important in patients receiving ICIs who develop new-onset hyperglycemia or compatible symptoms. Once DKA is diagnosed, immediate intravenous fluid resuscitation, continuous intravenous insulin infusion, and correction of electrolyte imbalance and acidosis are required to prevent severe metabolic complications. Decisions regarding continuation of immunotherapy should be individualized according to oncologic response and metabolic stability. In Case 1, PD-1 inhibitor therapy had already been discontinued because of disease progression before the onset of ICI-DM. In contrast, Case 2 achieved a partial tumor response and maintained acceptable glycemic control with insulin, allowing continuation of PD-L1 inhibitor therapy. These cases illustrate that the management of immunotherapy after ICI-related dysglycemia should be individualized and guided by both oncologic benefit and metabolic control (34). Regular glucose monitoring remains essential throughout ICI therapy and during follow-up after treatment discontinuation. Because delayed-onset ICI-DM may occur after cessation of immunotherapy, continued surveillance and patient education are clinically important. Blood glucose levels may remain within the normal range until shortly before onset and then rise rapidly, making periodic monitoring crucial for early detection (2). Although genetic susceptibility has been suggested, including possible associations with HLA genotypes, such as HLA-DR4, HLA typing was not performed in our patients; therefore, the contribution of genetic predisposition could not be assessed in this study. Future studies are needed to determine whether pre-treatment HLA genotyping may help identify individuals at increased risk for autoimmune endocrine adverse events and thereby support closer metabolic monitoring. Importantly, such risk stratification should aim to facilitate earlier recognition and reduce morbidity rather than preclude potentially beneficial immunotherapy (35).

## 4 Conclusion

ICI-DM is an underrecognized immune-related adverse event associated with immune checkpoint inhibitor therapy. Because its onset may be abrupt, its presentation may be non-specific, and it can occur even after treatment discontinuation, early recognition remains challenging. Continued blood glucose monitoring during and after immunotherapy is therefore important. When unexplained hyperglycemia occurs, ICI-DM should be considered. Early recognition and timely intervention may improve clinical outcomes without necessarily compromising antitumor treatment.

## Data Availability

The original contributions presented in the study are included in the article/supplementary material. Further inquiries can be directed to the corresponding author.
